# Epigenetic changes to gene pathways linked to male fertility in ex situ black‐footed ferrets

**DOI:** 10.1111/eva.13634

**Published:** 2024-01-26

**Authors:** Stavi R. Tennenbaum, Robyn Bortner, Colleen Lynch, Rachel Santymire, Adrienne Crosier, Jenny Santiestevan, Paul Marinari, Budhan S. Pukazhenthi, Pierre Comizzoli, Melissa T. R. Hawkins, Jesús E. Maldonado, Klaus‐Peter Koepfli, Bridgett M. vonHoldt, Alexandra L. DeCandia

**Affiliations:** ^1^ Ecology and Evolutionary Biology Princeton University Princeton New Jersey USA; ^2^ U.S. Fish & Wildlife Service National Black‐Footed Ferret Conservation Center Carr Colorado USA; ^3^ Riverbanks Zoo and Garden Columbia South Carolina USA; ^4^ Biology Department Georgia State University Atlanta Georgia USA; ^5^ Center for Species Survival Smithsonian's National Zoo and Conservation Biology Institute Front Royal Virginia USA; ^6^ Center for Animal Care Sciences Smithsonian's National Zoo & Conservation Biology Institute Front Royal Virginia USA; ^7^ Division of Mammals, Department of Vertebrate Zoology National Museum of Natural History Washington DC USA; ^8^ Center for Conservation Genomics Smithsonian's National Zoo and Conservation Biology Institute Washington DC USA; ^9^ Smithsonian‐Mason School of Conservation George Mason University Front Royal Virginia USA; ^10^ Biology Georgetown University Washington DC USA

**Keywords:** conservation, DNA methylation, infertility, *Mustela nigripes*, reproduction, sperm

## Abstract

Environmental variation can influence the reproductive success of species managed under human care and in the wild, yet the mechanisms underlying this phenomenon remain largely mysterious. Molecular mechanisms such as epigenetic modifiers are important in mediating the timing and progression of reproduction in humans and model organisms, but few studies have linked epigenetic variation to reproductive fitness in wildlife. Here, we investigated epigenetic variation in black‐footed ferrets (*Mustela nigripes*), an endangered North American mammal reliant on ex situ management for survival and persistence in the wild. Despite similar levels of genetic diversity in human‐managed and wild‐born populations, individuals in ex situ facilities exhibit reproductive problems, such as poor sperm quality. Differences across these settings suggest that an environmentally driven decline in reproductive capacity may be occurring in this species. We examined the role of DNA methylation, one well‐studied epigenetic modifier, in this emergent condition. We leveraged blood, testes, and semen samples from male black‐footed ferrets bred in ex situ facilities and found tissue‐type specificity in DNA methylation across the genome, although 1360 Gene Ontology terms associated with male average litter size shared functions across tissues. We then constructed gene networks of differentially methylated genomic sites associated with three different reproductive phenotypes to explore the putative biological impact of variation in DNA methylation. Sperm gene networks associated with average litter size and sperm count were functionally enriched for candidate genes involved in reproduction, development, and its regulation through transcriptional repression. We propose that DNA methylation plays an important role in regulating these reproductive phenotypes, thereby impacting the fertility of male ex situ individuals. Our results provide information into how DNA methylation may function in the alteration of reproductive pathways and phenotypes in artificial environments. These findings provide early insights to conservation hurdles faced in the protection of this rare species.

## 1 INTRODUCTION

Epigenetic variation can influence gene expression without altering the underlying genomic sequence. Such mechanisms are flexible and fast‐acting with respect to evolutionary time, can be influenced by an organism's environment (Consuegra & Rodríguez López, 2016; vonHoldt et al., 2022), can change within its lifetime (Fraga et al., 2005), and can potentially undergo transgenerational inheritance to offspring (Herman et al., 2016). While the effects of epigenetic variation have been shown to influence development (Skinner, 2011), reproduction (Stuppia et al., 2015), behavior (Jensen, 2013), stress tolerance (Hunter, 2012), and immunity (Morandini et al., 2016) in both humans and model organisms, these mechanisms have not been investigated in the context of wildlife health and conservation genetics, especially in ex situ managed species (DeCandia et al., 2018; Lamka et al., 2022; Rey et al., 2019; Zeng et al., 2022). As a result of environmental change (e.g., artificial settings), rapid and potentially (mal)adaptive epigenetic modifications to regulatory gene pathways may lead to phenotypic changes across more than one biological pathway that substantially decrease both individual and reproductive fitness.

DNA methylation is among the best characterized forms of epigenetic modifications in mammals (Bossdorf et al., 2008; Verhoeven et al., 2016) and can reversibly regulate gene expression through alternative splicing, post‐translational modifications, and silencing gene promoters (i.e., protein‐binding regions of the genome located upstream of genes where transcription is initiated) (Smith & Meissner, 2013). DNA methylation has been linked as both a cause and consequence to variation in gene regulation (Pacis et al., 2019), has been studied prolifically as a key biomarker for aging and disease for several decades (Mattei et al., 2022; Prado et al., 2021), and is associated with variation in reproductive metrics in humans, model laboratory organisms, and commercially relevant species (Rotondo et al., 2021; Sujit et al., 2020). Recent studies have identified an increasingly important role for DNA methylation variation in sperm to fertility metrics in the ex situ breeding of domesticated animals (Costes et al., 2022; Štiavnická et al., 2022; Takeda et al., 2021).

In the current era of global climate change, ex situ breeding of wildlife is a powerful tool for conserving at‐risk species. Serving as modern‐day “Arks,” animal populations under human management provide a safety net for threatened species when extirpation or extinction in the wild may be imminent (Minteer et al., 2018). While ex situ breeding has helped numerous species survive population decline, human management can also have unexpected negative consequences due to low number of founders, small population size, erosion of genetic diversity, or negative responses to artificial settings (Lacy, 2013). Some individuals may even exhibit evidence of inbreeding depression or reduced fitness following sustained consanguineous mating (Willoughby et al., 2017; Willoughby & Christie, 2019). Although this phenomenon is traditionally attributed to loss of genetic diversity, disparities often exist between the health and reproductive capacity of ex situ individuals and individuals born in the wild from reintroduced parents. This suggests that inbreeding depression may not derive solely from demographic history and could have a compounding environmental component—a phenomenon known as environmentally determined inbreeding depression (EDID) (Cheptou & Donohue, 2011). Major phenotypic variability between genetically similar populations due to environmental differences (as observed in EDID) could be explained in part by epigenetic mechanisms (Cheptou & Donohue, 2013; Dapp et al., 2015; Vergeer et al., 2012).

The black‐footed ferret (*Mustela nigripes*; hereafter, BFF) is an ideal species in which to investigate the possibility for an epigenetic basis to EDID due to its extremely low genetic variation resulting from a severe population bottleneck, long history of ex situ management, extensive availability of biobanked samples, and detailed accounts of individual and reproductive fitness (Santymire, 2016). Once believed to be extinct, BFFs were rediscovered in Meeteetse, Wyoming in 1981 and brought into managed care in the late 1980s when low prey density and disease threatened to decimate remaining individuals (USFWS, 2013; USGS et al., 2005). From seven genetic founders, over 10,500 kits have been born in the last three decades in the ex situ population, with roughly 650 animals currently sustaining the species across ex situ propagated and reintroduced populations (Marinari & Lynch, 2022). Ongoing conservation efforts include pedigree‐based breeding in ex situ BFFs and reintroduction to areas of their native range (USFWS, 2013; USGS et al., 2005), with a recent push toward conservation cloning to resurrect pre‐bottleneck genetic variation and gene editing to encode disease resistance (Sandler et al., 2021; Wisely et al., 2015). While some of these efforts suggest a conservation success story, genetic diversity is severely limited in both the reintroduced and ex situ managed populations due to extreme founder effects (Wisely et al., 2002, 2008).

Ex situ BFFs now exhibit increasing physiological evidence of inbreeding depression, including poor sperm quality (teratospermia) in males and low whelping success in females (Santymire et al., 2014, 2019; Wisely et al., 2002, 2008). While such problems may be due to their genetic history (Lawrence et al., 2017), dietary changes (Santymire et al., 2020), or stress (Santymire et al., 2021), conservation managers have reported that males specifically experience strong variation in reproductive success dependent on rearing environment: wild‐born progeny descended from ex situ males exhibit significantly improved seminal traits (Rachel Santymire, pers. comm.). Furthermore, up to 50% of ex situ bred males between 1 and 3 years old (the prime reproductive age range for males in the wild) fail to sire offspring in dedicated breeding facilities (Wolf, Wildt, Vargas, Marinari, Ottinger, & Howard, 2000). This difference suggests that environmental variation between wild and artificial conditions may be the key component driving this divergence in reproductive capacity. Previous work on ex situ managed BFFs and their reproductive decline under human care has focused on sperm, as seminal traits have been linked to the notable declines in fecundity observed ex situ via decreased pregnancy rates and litter sizes (Pukazhenthi et al., 2007; Pukazhenthi & Wildt, 2004; Santymire et al., 2019), but some studies have shown that seminal traits are not the primary predictor of reproductive inefficiency in male BFFs (Wolf, Wildt, Vargas, Marinari, Kreeger, et al., 2000; Wolf, Wildt, Vargas, Marinari, Ottinger, & Howard, 2000).

We hypothesized here that epigenetic modifications to DNA, specifically DNA methylation (i.e., the addition of a CH_3_ methyl group to cytosine base pairs often occurring within the CpG context), may underlie the decline in reproductive capacity observed in ex situ facilities. Specifically, we hypothesized that changes in DNA methylation in promoters or in gene bodies involved in reproductive and developmental processes would be associated with fertility changes in ex situ bred BFFs. To test these hypotheses, we generated a novel epigenetic dataset and leveraged uniquely available long‐term phenotypic data from BFFs bred in human care. We selected male individuals that were born in ex situ facilities and reared as potential breeders, had biobanked samples representing at least two different tissue types, and varied in their lifetime reproductive metrics to explore the link between reproductive fitness and DNA methylation. Detailed life‐history data are archived for all ex situ bred BFFs; this information allowed us to account for variables known to impact DNA methylation variation, such as relatedness and age (Laine et al., 2022; Lea et al., 2016), and focus specifically on the relationship between DNA methylation and male reproductive metrics.

We used reduced representation bisulfite sequencing (RRBS) to conduct the first survey of DNA methylation across the genome of ex situ managed BFFs. We profiled DNA methylation in three tissues with plausible reproductive relevance: whole blood, testes, and sperm, each of which is regularly collected and banked during biomedical surveys of possible breeders. Biobanking of reproductive tissues is foundational to national BFF conservation efforts (Santymire, 2016) and therefore provided us with an opportunity to study tissues that are rarely profiled or compared in DNA methylation studies in wildlife. We surveyed these tissues for genome‐wide DNA methylation variation associated with three male reproductive phenotypes: average litter size (for all tissues), sperm count per mL for sperm, and tissue firmness for testes (which indicated breeding readiness). We used average litter size as a proxy for lifetime reproductive success, sperm count per mL as a measure of the current reproductive condition of an individual (since DNA methylation can vary through time), and testes firmness as a representative measure of seasonal variation between breeding and non‐breeding seasons.

In this study, we aimed to contribute a novel perspective to ex situ population management and are among the first to survey epigenetic variation in ex situ managed wildlife in the context of reproductive health and fitness. By surveying three distinct tissue types, we identified the most promising sample type for associating patterns of DNA methylation to important fecundity metrics that range from ephemeral (sperm count) to seasonal (testes firmness) to lifetime averages (average litter size). We associated DNA methylation variation with these distinct reproductive phenotypes and explored patterns of differential methylation at genomic CpGs in addition to their role within larger gene networks. Considered together, these analyses allowed us to assess the functional role of DNA methylation within these networks and evaluate their relevance to reproductive biology in BFFs. This study provides insight into potential epigenetic mechanisms underlying the declining reproductive health of an endangered species and establishes critical baseline data for further study of molecular changes that may hamper efforts to bring species like the BFF back from the brink of extinction.

## 2 METHODS

### 2.1 Sample collection and DNA extraction

We obtained all samples used in this study from the BFF biobank maintained at the Smithsonian's National Zoo and Conservation Biology Institute: eight whole blood samples from venipunctures carried out regularly in ex situ facilities, nine testes samples collected via dissection during castration, and five semen samples collected via standard electroejaculations used for artificial insemination attempts (Howard et al., 1991). These samples were derived from nine male ex situ BFFs sampled between 2012 and 2020 (see Table 1), with at least two out of three representative sample types taken from all individuals and testes samples taken from all nine individuals. Reproductive data from these animals are collected annually as part of standard protocol at the National Black‐footed Ferret Conservation Center, outlined in the Managed Care Operations Manual implemented as part of the Black‐footed Ferret Species Survival Plan® (Marinari & Lynch, 2022). We derived metadata and reproductive phenotypes for selected samples from the BFF studbook, including animal identifier, date of sample collection, date and location of birth, number of litters (annual and lifetime total), litter size (annual and lifetime average), and date and cause of death. Other reproductive metrics such as pairing response behavior and success, reproductive readiness, and testes firmness are collected through observation and routine sampling performed by animal keepers and breeding managers.

**TABLE 1:** The twenty‐two samples used in this study derived from nine male ex situ black‐footed ferrets.

Sample ID	Studbook No.	Name	Tissue type	Collection date	Age at sampling	Lifetime average litter size	Testes status at collection	EEJ sperm count per mL (in millions)
B2_2	7611	Gale	Blood	11_29_2017	4	0	Not_Firm	n/a
B3_1	7419	Tanis	Blood	04_11_2018	6	2	Firm	n/a
B4_1	8519	Jensen	Blood	12_12_2017	2	0	Not_Firm	n/a
B5_2	8520	Padalecki	Blood	04_10_2018	3	2	Firm	n/a
B6_2	7685	Cane	Blood	08_08_2018	5	4.5	Not_Firm	n/a
B7_2	7916	Eamon	Blood	12_04_2017	4	3.8	Not_Firm	n/a
B8_1	8161	Flagstaff	Blood	12_05_2017	3	4.3	Not_Firm	n/a
B9_1	8393	Pancake	Blood	05_09_2018	3	0	Firm	n/a
S1_1	6536	Capone	Sperm	03_27_2012	3	1.5	Firm	407.1
S2_2	7611	Gale	Sperm	04_25_2017	4	0	Firm	1018.4
S3_2	7419	Tanis	Sperm	04_11_2018	6	2	Firm	572.1
S4_1	8519	Jensen	Sperm*	04_5_2019	4	0	Firm	69.8
S5_1	8520	Padalecki	Sperm	04_10_2018	3	2	Firm	888.2
T1_2	6536	Capone	Testes	05_27_2015	6	1.5	Firm	n/a
T2_1	7611	Gale	Testes*	02_13_2018	5	0	Firm	n/a
T3_2	7419	Tanis	Testes	04_11_2018	6	2	Firm	n/a
T4_1	8519	Jensen	Testes	08_01_2019	4	0	Not_Firm	n/a
T5_1	8520	Padalecki	Testes	10_25_2018	3	2	Not_Firm	n/a
T6_1	7685	Cane	Testes	08_10_2018	5	4.5	Not_Firm	n/a
T7_1	7916	Eamon	Testes	05_02_2018	5	3.8	Firm	n/a
T8_2	8161	Flagstaff	Testes	01_16_2020	6	4.3	Firm	n/a
T9_2	8393	Pancake	Testes	05_10_2018	3	0	Firm	n/a

*Note*: Studbook No. and name denote the individual's studbook number and given name according to the Association of Zoos and Aquariums Black‐footed Ferret Species Survival Plan® managed pedigree; sample ID, tissue type, collection date, and age at sampling provide details about the banked samples used in this study; and lifetime average litter size, testes status at collection, and electroejaculation (EEJ) sperm count per mL (in millions) provide information about the reproductive phenotypes used in this study. Tissue type asterisks denote RRBS replicates.

Due to BFF management practices that influence demography and breeding opportunity, ex situ male reproductive success is well‐documented and differs from the observed reproductive capacity of wild individuals. Over the course of BFF population management under human care (1985–2022), mean lifetime offspring per proven male litter sired is 15.29 (range 1–105), median lifetime number of offspring per litter sired is 11, and mean litter size is 4.18, suggesting a lifetime observed average litter size of 3.66 for breeding males (Marinari, 2022). However, we did not investigate lifetime number of litters as a proxy for reproductive success, as four of the males studied here (7611: Gale; 8519: Jensen; 8520: Padalecki; and 8393: Pancake) never had successful natural matings due to their aggression toward estrous females: their only lifetime litters resulted from artificial insemination. Therefore, these males would have had fewer attempts to sire litters than males able to pair naturally. Additionally, males are paired with different females that further vary in reproductive quality throughout their lifetime. As we aimed to investigate the reproductive capacity and contribution of ex situ males specifically, we used average litter size (number of kits per litter) to control for differences in female partners and number of lifetime pairing opportunities.

We obtained testes firmness data for the nine testes samples used in this study as a seasonal variable, as testes firmness is evaluated by physical palpitation several times per month throughout the breeding season prior to castration (roughly January to June). We also obtained sperm count per mL as a metric of male fertility at electroejaculation for the five semen samples. This electroejaculation procedure was developed for BFFs to maintain seminal quality criteria (Howard, 1993; Howard et al., 1986, 1991, 2016; Santymire et al., 2004, 2006, 2007; Wildt et al., 1989). Briefly, BFFs are anesthetized using a 40 mg/kg of ketamine/diazepam mixture. A pattern of stimulations then results in ejaculation and semen is collected from the glans penis using a micropipette (Howard, 1993; Wildt et al., 1989). Semen volume was measured and transferred immediately into 100 μL of warmed (37°C) TEST‐yolk buffer (TYB; Irvine Scientific, Santa Ana, CA, USA) for cryopreservation (Wolf, Wildt, Vargas, Marinari, Kreeger, et al., 2000). All samples selected for this study were previously collected during routine management activities, banked, and released for research in November 2020. All animals were sampled according to the Guide for Care and Use of Laboratory Animals, approved by the Lincoln Park Zoo Research Committee (Chicago, IL) and the U.S. Fish and Wildlife Service (Carr, CO).

Genomic DNA was extracted from all three tissue types using the DNeasy Blood and Tissue Kit following the standard manufacturer's protocol (Qiagen, Inc). To lyse sperm cells within semen samples (hereafter referred to as sperm), we followed a modified version of the user‐developed protocol “Purification of total DNA from animal sperm using the DNeasy Blood & Tissue Kit; protocol 2” made available by Qiagen in August 2003. Briefly, we first prepared 1 mL of buffer X2 by thoroughly mixing the following reagents: 20 μL 1 M Tris‐Cl (pH 8.0), 40 μL 0.5 M EDTA, 40 μL 5 M NaCl, 400 μL 10% SDS, and 500 μL molecular grade water. We combined 100 μL of sperm with 100 μL buffer X2 and then added 8 μL 1 M DTT and 20 μL Proteinase K to each mixture to incubate overnight at 56°C while mixing at 500 rpm. We then followed the standard manufacturer's protocol to complete the genomic DNA extraction from the digested sperm cells. We eluted all samples twice, using 50–80 μL buffer AE pre‐heated to 70°C and incubated at room temperature for 10 min (first elution) and 20 min (second elution). We reduced sample volume in an Eppendorf Vacufuge (set to V‐AQ 45°C), cleaned each sample using a ratio of 1 DNA:2.4 Agencourt AMPure XP magnetic beads (Beckman Coulter, Indiana, USA), and eluted in a final volume of 30–45 μL 0.1X TE to concentrate DNA extracts. DNA extracts were quantified using Qubit fluorometry (Thermo Fisher Scientific, Waltham, MA, USA).

### 2.2 RRBS library preparation

We prepared 1 μg of genomic DNA for RRBS and spiked each sample with 1 μL of lambda DNA to enable calculations of bisulfite conversion efficiency. We then prepared methylation libraries following a protocol modified from Meissner et al. (2005), based on the NEBNext Multiplex Oligos for Illumina Methylated Adaptor (NEB #E7535) instruction manual v2.0. We digested genomic DNA with the restriction enzyme *Msp1* (which selects for DNA fragments that begin and/or end with a CpG dinucleotide, e.g., CpG islands) before repairing the 3′ ends with adenine, ligating methylated adaptors, and size selecting to retain fragments 200–350 bp in length using a ratio of 1 DNA:0.55 Agencourt AMPure XP magnetic beads, followed by a ratio of 1 DNA:0.16 Agencourt AMPure XP magnetic beads. We performed bisulfite conversion of unmethylated individual cytosines following the Qiagen EpiTect Fast DNA Bisulfite Kit manufacturer's protocol, subsequently enriched for adaptor‐ligated fragments using 15 cycles of PCR, and uniquely barcoded each library using NEBNext Index Primers. We performed a double bead clean of PCR products using a ratio of 1 DNA:0.9 Agencourt AMPure XP magnetic beads, followed by a ratio of 1 DNA:1 Agencourt AMPure XP magnetic beads, and eluted with 33 μL 0.1X TE. We pooled 50 ng of each sample to construct our final pooled libraries, which were then concentrated with a final bead clean. Each library pool consisted of 12 samples, with two samples duplicated across libraries to serve as positive controls. Whole blood, testes, and sperm samples were evenly distributed between libraries, along with samples obtained from each individual BFF to minimize potential batch effects (Table S1). We sequenced the pooled libraries on a NovaSeq 6000 SP (1 × 100 nt) flow cell at the Princeton University Lewis Sigler Genomics Core Facility.

### 2.3 Bioinformatic processing

We demultiplexed single‐end reads in each sequencing library, with no mismatches allowed between the observed and expected barcode sequences. We used *cutadapt* v1.8.1 (Martin, 2011) to trim low‐quality bases (*−q* 20), clip adaptor sequences from the 3′ end of each demultiplexed read, and discarded reads shorter than 20 nucleotides in length. We then used *BS‐Seeker2* (Guo et al., 2013) and *bowtie2* (Langmead & Salzberg, 2012) to build an RRBS index with 50–500 bp fragments from the domestic ferret reference genome *MusPutFur1.0* (Peng et al., 2014; NCBI BioProject PRJNA59869) using *bs_seeker2‐build.py*. We mapped sequence data to this indexed reference using *bs_seeker2‐align.py* and estimated cytosine methylation levels with 1× and 10× minimum coverage using *bs_seeker2‐call_methylation.py*. Matching sequenced cytosines between the bisulfite‐converted reads and reference sequences indicated methylated sites, whereas cytosine‐to‐thymine transitions indicated unmethylated cytosines (Meissner et al., 2005). We additionally mapped reads to the lambda linear genome (NC_001416.1) to calculate bisulfite conversion efficiency by subtracting the average methylation frequency across the lambda genome from one for each sample. Quality‐filtered reads were used to explore overall methylation frequency and the genomic distribution of individually sequenced, methylated cytosines at three different CpG methylation motifs across the BFF genome. As many studies have confirmed that DNA methylation in most vertebrate tissues occurs predominantly in the CG dinucleotide motif (Ross et al., 2020; Suzuki & Bird, 2008), including mammalian sperm (Molaro et al., 2011; Štiavnická et al., 2022) and blood (Derks et al., 2016), we used only the CG motif methylation in all downstream analyses that reference CpG sites.

### 2.4 Differential DNA methylation

We formatted CGmap files for use in the *R* package *methylKit* (Akalin et al., 2012). We filtered bases for a minimum of 10X coverage and united all samples into a single file that retained nucleotides with adequate sequence coverage across all specified samples. We implemented principal component analysis in *methylKit* to determine similarity between positive control samples and retained the replicate sample with higher bisulfite conversion efficiency. We calculated the methylation frequency (MF) per cytosine as the proportion of methylated cytosines out of the total number of methylated and unmethylated cytosines (sequenced as thymine) reads per site. We then created separate datasets for each sample type, yielding four datasets in total (i.e., the full 22‐sample dataset, the whole blood dataset (*n* = 8), the testes dataset (*n* = 9), and the sperm dataset (*n* = 5)). For each dataset, we performed principal component analysis (PCA) of methylation frequency using the *R* core function *prcomp* and visualized results using the *R* package *ggplot2* (Wickham, 2016).

For each tissue type, we identified differentially methylated sites (DMS) associated with male reproductive phenotypes using binomial mixed model regression in MACAU (Lea et al., 2015), a flexible framework which uniquely accounts for the over‐dispersed and count‐based nature of RRBS data and improves the power of detecting DMS over a linear or beta‐binomial model approach. This model was custom developed for and validated using both simulated and real RRBS and whole‐genome bisulfite data. MACAU provides well‐calibrated test statistics in the presence of population structure—an especially important variable in small, closely related populations such as ex situ BFFs. To detect differentially methylated sites, MACAU models each potential target of DNA methylation individually (i.e., each CpG site is modeled independently) as a function of X, an environmental predictor variable of interest (Lea et al., 2016). Here, we used (1) average litter size, (2) sperm count per mL, and (3) testes firmness as our independent predictor variables of male fertility and included sample covariates to control for shared ancestry between male BFFs (i.e., pedigree‐based relatedness estimates) and age (i.e., age at sample collection). We then ran a total of five separate analyses using this model: (1) blood and average litter size; (2) testes and average litter size; (3) sperm and average litter size; (4) sperm and sperm count per mL; and (5) testes and testes firmness, where the independent variables were again average litter size, sperm count, and testes firmness.

We assessed *p*‐value distributions from binomial mixed models using quantile–quantile plots for each of our five analyses. We found deviation from statistical expectations in models for three out of five analyses: evidence for p‐value deflation in our sperm analyses, *n* = 5, and modest *p*‐value inflation in one testes analysis, *n* = 9 (Figure S6). Note that a uniform distribution of *p*‐values may not be a reasonable assumption for our tissue‐specific datasets (i.e., sperm and testes), in which we can expect that most cells are engaged in a small, targeted set of biological processes, possibly leading to a bias of values above or below the uniform distribution. While these modeled *p*‐values deviated from uniform expectations, this may indicate real biological variation in the global prevalence of methylated genomic sites associated with reproductive phenotypes in these specialized tissues. However, we ultimately cannot conclude that sample size alone did not generate these deviations. Due to the large number of candidate DMS produced from binomial mixed models, we adopted a distribution‐based approach to focus downstream efforts only on the most extreme outliers in probability of methylation and significance as detected by MACAU. We designated significant outliers from models as cytosines with beta (*ß*) values in the 1st or 99th percentiles of the distribution and *p*‐values in the lowest 1st percentile, as in vonHoldt et al. (2022). Lastly, we annotated significant DMS as either intergenic, intronic, exonic, or within 2 Kb of a transcription start site (i.e., within promoters) in the domestic ferret genome (Agirre et al., 2015).

First, we assessed whether annotated cytosines associated with male reproductive phenotypes from MACAU were functionally enriched for specific gene ontological (GO) categories using the program g:Profiler. Our annotated gene IDs served as in‐files for GO analyses run in *g:gost* in *R* (Raudvere et al., 2019). We searched all available annotations for molecular function (MF), cellular component (CC), and biological process (BP) in addition to open‐source databases for the domestic ferret *mpfuro* (i.e., KEGG, Reactome, WikiPathways, TRANSFAC, miRTarBase, Human Protein Atlas, CORUM, and Human Phenotype Ontology). We used a Benjamini–Hochberg FDR cutoff of 0.05 to determine GO enrichment in a category (Benjamini & Hochberg, 1995). We used the *R* package *VennDiagram* to identify overlapping outlier sites and GO terms associated with each of our five association tests (Chen & Boutros, 2011).

To further identify and profile larger, functional stretches of differential methylation in the BFF genome, we identified differentially methylated clusters (DMclusters) by grouping all significant DMS within a physical inter‐cytosine distance of <40 bp which fell within annotated regions of the domestic ferret genome, as in vonHoldt et al. (2022). We acknowledge that there is inherent bias in analyzing physical clustering of cytosines given the reduced representation method of fragment selection in RRBS. Our goal in these analyses was to capture information at the single DMS and at the larger regional level of methylation in the genome for more meaningful functional profiling via gene interaction networks.

### 2.5 Gene network functional enrichment

We used annotated DMclusters containing three or more contiguous, significant DMS (Lea et al., 2015) as input to construct gene interaction networks from our list of annotated domestic ferret gene IDs, converted in Ensembl *biomaRt* (Cunningham et al., 2022). We used the *STRING* plugin v1.4.2 (Szklarczyk et al., 2019) in *Cytoscape* v3.9.1 (Shannon et al., 2003), which uses databased and computationally predicted domestic ferret protein interactions from gene expression data to assemble gene interaction networks (Cline et al., 2007). We used a confidence cutoff of 0.7 and viewed all network interactors to evaluate known connectivity between genes for each of our five separate analyses (Lindner et al., 2021). To explore whether constructed gene networks themselves were enriched for functional terms with biological relevance to reproduction, we then retrieved the functional enrichment information from Cytoscape for these networks, which included candidate DMS previously identified from our differential methylation analysis. We imported all directly connected gene interaction terms to g:Profiler2 to identify their regulatory importance and possible biological functions in mammalian pathways (Lindner et al., 2021; vonHoldt et al., 2022). We used the same *g:gost* settings described previously to search for all known gene annotations in the *Mus musculus* genome, as both domestic ferrets and BFFs have comparatively less publicly available annotated genomic data. After extracting the top enriched GO terms for each of our five separate gene networks, we focused a final effort on our two sperm analyses that examined differential DNA methylation in association with average litter size and sperm count per mL, as these analyses proved the most informative for linking epigenetic variation to male reproductive phenotypes. We then investigated all genes and pathway‐specific functions annotated by our most significant GO terms for each analysis in the Ensembl (Cunningham et al., 2022) and OMIM databases (Hamosh et al., 2002).

## 3 RESULTS

### 3.1 DNA methylation sequencing and annotation

We characterized epigenetic variation at cytosines within CG‐enriched regions using a reduced representation methylation profiling approach across the BFF genome. We obtained a total of 906,277,226 raw sequences from our 24 methylation libraries. Demultiplexed reads per sample ranged from 28,510,542 to 45,664,513, with an average ± SD of 35,909,062.00 ± 4,282,576.17 (Table S1). We mapped sequence reads to the domestic ferret genome and filtered for a minimum of 10× coverage, retaining 13,581,877 to 24,299,800 CpG sites per sample (18,843,903 ± 2,749,421) on average after 10× filtering (Table S2). Samples had an average coverage of 24.4 ± 2.2 and bisulfite conversion rate of 99.4% ± 0.1% (Table S2). Average methylation frequency was 0.10 ± 0.01 across all cytosines, with much higher average methylation observed in CG motifs (0.50 ± 0.04) compared to CHG and CHH motifs for all tissues (both 0.003 ± <0.001; Figure S1), where *H* = A, T, C. Due to very low instances of non‐CG methylation in the three tissues analyzed, we excluded results from CHG and CHH motifs and used the CG motif as the only CpG dinucleotide included in all subsequent methylation analyses. The two positive control samples we replicated across sequenced libraries showed highly similar summary statistics (Table S2) and clustering in PC space (Figure S2). Thus, we retained the replicate with higher conversion efficiency (S4_1 and T2_1) for downstream analyses of 22 unique samples.

### 3.2 DNA methylation at genomic CpGs shows distinct clustering by tissue type

**FIGURE 1:**
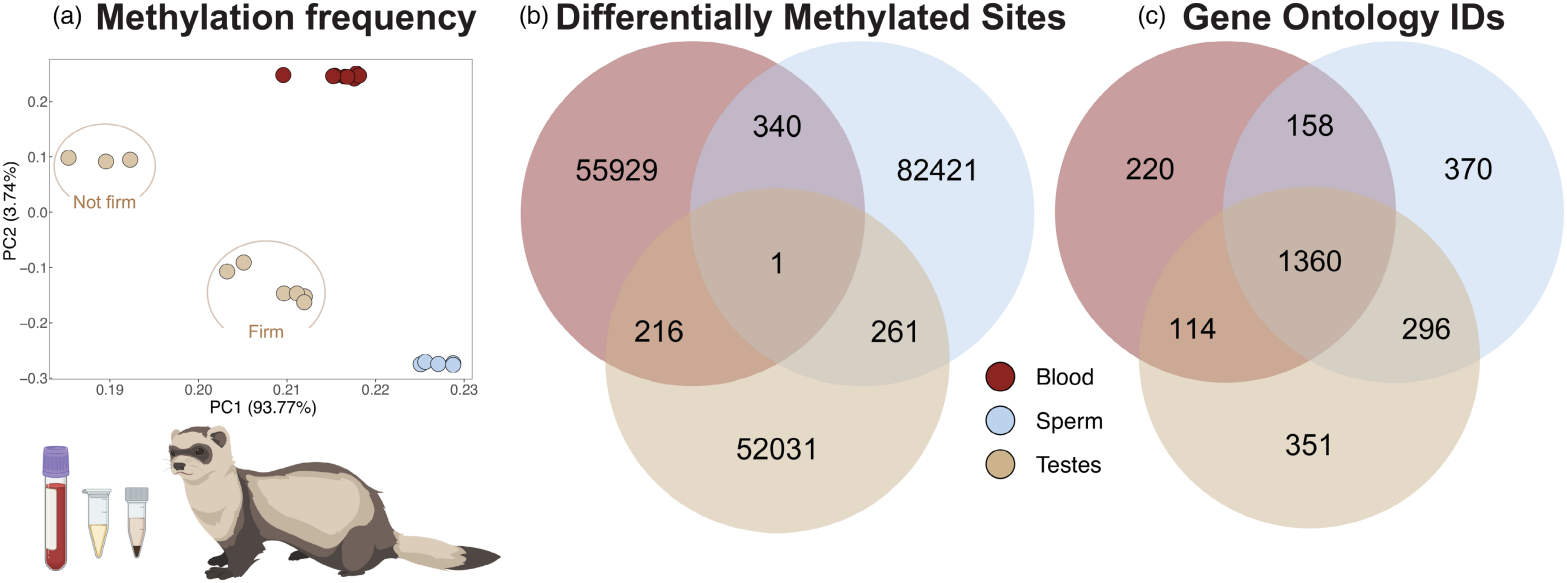
Relationship between tissue type, genome‐wide patterns of DNA methylation, and the reproductive phenotype of average litter size in male BFFs. (a) Principal component analysis of methylation frequency by tissue type for all samples (*n* = 22), including testes collected both during and outside the breeding season. (b) Tissue‐type specificity in DMS that reached significance (cytosines with beta (ß) values in the 1st or 99th percentiles of the distribution and p‐values in the lowest 1st percentile) in three tissue types tested for association with average litter size. (c) Overlap in Gene Ontology enrichment terms from significant DMS in all three tissues tested for association with average litter size.

Principal component analysis of methylation frequency revealed clustering by sample type along the first four PCs (Figure 1a, Figure S3). To explore additional drivers of sample clustering, we tested for associations between the first five PCs and the variables sample type, ferret ID, batch ID, and bisulfite conversion rate. Sample type was significantly associated with the first four PCs (Kruskal–Wallis test, df = 2; *p* < 0.001 for PC1–PC3; *p* = 0.042 for PC4) and showed distinct clustering across each PCA plot (Figure 1a, Figure S3), and bisulfite conversion rate was significantly associated with PC1 (Pearson's correlation, *t* = 2.088, df = 20, *p* = 0.050) and PC3 (*t* = −2.994, df = 20, *p* = 0.007) (Table S3). We observed no significant relationships between PC1 and PC5 with ferret ID (df = 8, *p* > 0.05) or batch ID (df = 1, *p* > 0.05). Due to the significant differences between sample types, we performed all subsequent analyses for each unique tissue type (blood, *n* = 8; testes, *n* = 9; sperm, *n* = 5). Notably, we observed PC sub‐clustering within testes samples linked to breeding season (Figure 1a, Figure S3). The three samples collected outside of the breeding season (T4, T5, and T6, all of which were phenotypically “not firm” upon collection) form a distinct cluster away from the six samples collected during the breeding season, all of which were phenotypically “firm” upon collection (Figure 1a).

### 3.3 DNA methylation is associated with reproductive phenotypes across tissue types

We conducted a binomial mixed model regression analysis and identified several million DMS associated with average litter size from our three tissue types (blood = 9,427,357; testes = 9,118,485; and sperm = 8,936,345), sperm count per mL (sperm = 9,494,233), and testes firmness (testes = 7,616,790; Table S4). Significance of DMS was determined using a two‐tailed distribution with two criteria: selecting beta (ß) values in the 1st or 99th percentiles of the distribution and p‐values in the lowest 1st percentile. After filtering by this dual threshold, blood analyses for average litter size yielded 56,328 DMS. Testes analyses for average litter size and testes firmness yielded 52,354 and 36,065 DMS, respectively. In contrast, sperm‐based analyses for average litter size and sperm count per mL yielded 82,776 and 84,325 significant DMS outliers, respectively. Across our five separate analyses, (1) blood and average litter size, (2) testes and average litter size, (3) sperm and average litter size, (4) sperm and sperm count per mL, and (5) testes and testes firmness, the majority of annotated DMS were located within promoters or gene bodies (69.5%) with the largest proportion of DMS (39%) occurring within introns (Figure S4). Although only one outlier DMS overlapped between all three tissue types (Figure 1b), we observed 1360 overlapping functional enrichment terms among blood, testes, and sperm datasets in association with average litter size, the phenotype tested across all three tissue types (Figure 1c).

### 3.4 Gene networks are enriched for development, differentiation, and morphogenesis

We identified thousands of significant, annotated DMclusters that met our DMS inter‐cytosine threshold of <40 bp: 30,373 DMclusters for blood and average litter size; 44,199 for sperm and average litter size; 28,727 for testes and average litter size; 43,261 for sperm count per mL; and 12,140 for testes firmness (Table S4). We created gene networks for each of these five analyses that associated tissue‐specific DNA methylation within significant DMclusters with three male reproductive phenotypes. Since single gene ID hubs within networks did not show high connectivity between nodes, we queried the functional enrichment for all directly connected gene interactors within each network (Figure S5). The gene network built from DMclusters containing three or more DMS from blood and average litter size (*n* = 414 gene IDs; Figure S5A) did not have any outliers associated with known reproductive processes. In contrast, gene networks built from DMclusters containing three or more DMS from testes (*n* = 449 gene IDs for testes and average litter size; *n* = 1327 gene IDs for testes firmness; Figure S5A) identified diverse terms associated broadly with system‐specific development (e.g., neuron projection and circulatory system regulation) and general cell morphogenesis, in addition to testes‐specific transcription factors (*MOVO‐B*).

**FIGURE 2:**
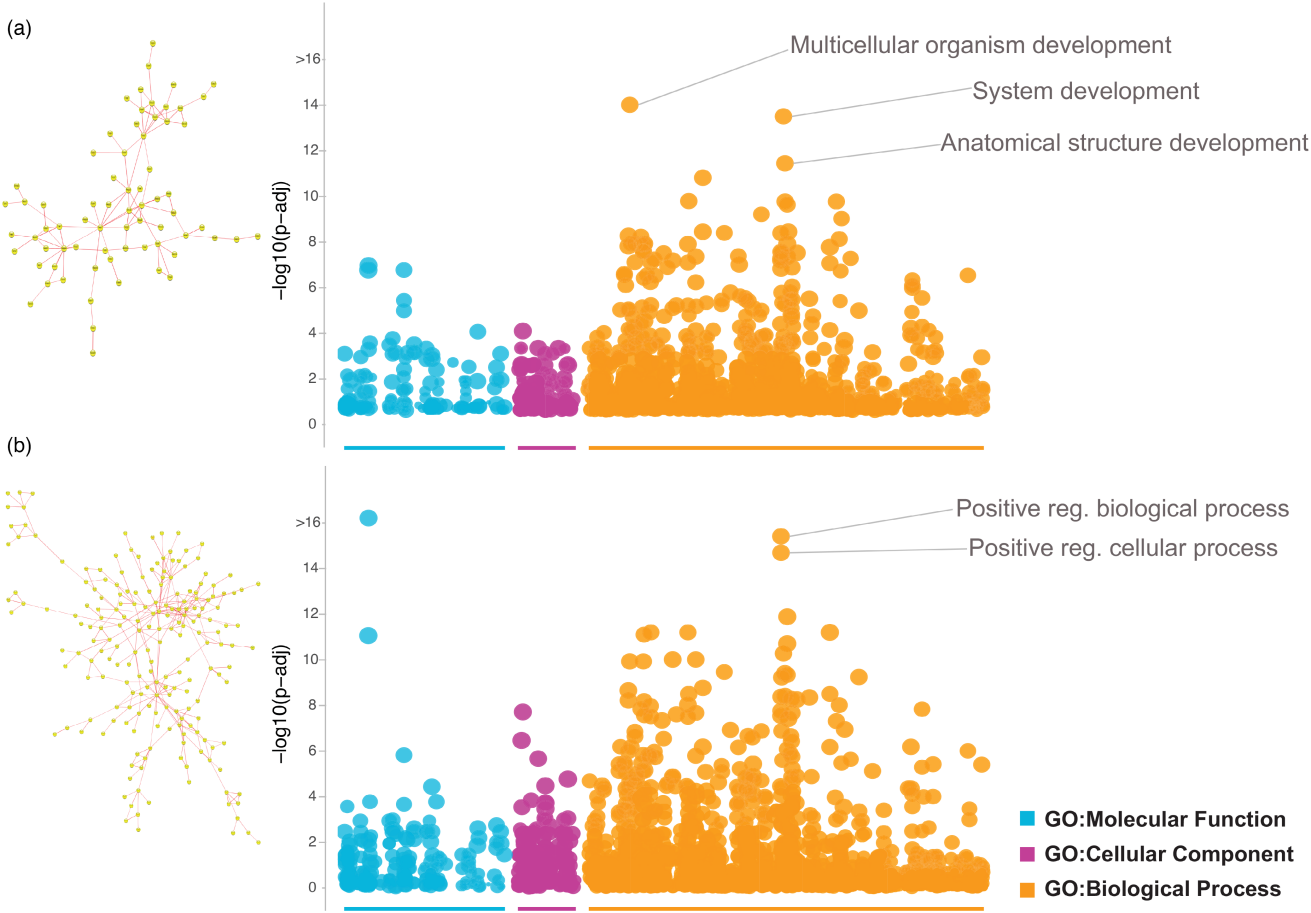
Gene networks built from significant DMclusters and respective GO enrichment plots for two analyses of the functional role of differential DNA methylation variation in sperm of ex situ BFFs. (a) Gene network structure and functional enrichment plot for DMclusters associated with variation in average litter size. (b) Gene network structure and GO functional enrichment plot for DMclusters associated with variation in sperm count per mL in male BFFs.

Gene networks built from DMclusters containing three or more DMS from sperm (*n* = 695 genes for sperm and average litter size; *n* = 948 genes for sperm count per mL) had outlier terms directly associated with reproduction and developmental biological processes (Figure 2). The top annotated genes were linked to multicellular organism development, system development, anatomical structural development and morphogenesis, positive regulation of developmental processes, and positive regulation of cellular processes (including cellular development). For our gene network analysis of sperm and average litter size, 49 genes were annotated by our top outlier GO term (GO:0048518; multicellular organism development; *p* = 3.27E−20). Five of these genes showed direct connections to reproductive regulatory processes, either in the regulation of development or differentiation through transcriptional repression (*EZH2*, *JARID2*; Table 2), or in putative functions related to morphogenesis of cells, tissues, and whole‐system differentiation (*PRKCZ*, *ADAM12*). In our gene network analysis of sperm count per mL, 128 genes were annotated by our top outlier GO term (GO:0048518; positive regulation of biological process; *p* = 6.59E−37), two of which are implicated in system‐specific development and differentiation (*EZH2*, *CAV3)*, and one of which was shared in our gene list from sperm and average litter size (*EZH2*; Table 2).

**TABLE 2:** Significant gene names and gene IDs annotated by Gene Ontology (GO) enrichment terms from gene network functional enrichment analyses of sperm, where “*” indicates average litter size and “^+^” indicates sperm count per mL.

Gene name	Gene ID	N GO terms	General functional role
Androgen receptor	AR*	417*	Steroid receptor; androgen insensitivity
ADAM metallopeptidase domain 12	ADAM12*	125*	Early muscular differentiation
Jumonji and AT‐rich interaction domain containing 2	JARID2*	303*	Transcriptional repression; cellular differentiation; and morphogenesis
Enhancer of zeste 2 polycomb repressive complex 2 subunit	EZH2*^+^	462*, 264^+^	Transcriptional repression; central nervous system development
Protein kinase C zeta	PRKCZ*	532*	Epithelial morphogenesis
Caveolin 3	*CAV3* ^+^	206^+^	Muscle development

## 4 DISCUSSION

We examined the role of DNA methylation in fertility in ex situ managed BFFs, an endemic North American mammal that has been the focus of intensive conservation management since its recent population bottleneck in the 1980s. Amid this backdrop of low genetic variation, conservation managers have observed reproductive declines in ex situ environments, evidenced by differences in the reproductive health of males (Santymire et al., 2019). This documented trend suggests that low genetic diversity may not be the only driver of male fertility decline and that environmentally determined inbreeding depression, or EDID, may play a role in the reproductive metric variation seen in ex situ managed individuals. Thus, we hypothesized that epigenetic changes to gene regulatory pathways through DNA methylation may underlie this manifestation of declining reproductive fitness in ex situ settings. To test this hypothesis, we characterized genome‐wide patterns of DNA methylation in male ex situ bred BFFs across three distinct tissue types (i.e., whole blood, testes, and sperm) that could yield changes in DNA methylation in or near gene networks with functional relevance to reproductive physiology.

We first surveyed these three tissues to determine which sample types provided the greatest insight into male BFF reproductive capacity and its ongoing decline in ex situ settings. We identified significant associations between DNA methylation and reproductive phenotypes across three scales: sperm count per mL, seasonal testes firmness, and lifetime average litter size. Through these analyses, we both characterized tissue‐specific DNA methylation and provided the first association of epigenetic variation to individual reproductive metrics in BFFs. These data provide critical baseline information on epigenetic mechanisms relevant to organismal fitness in this species. To our knowledge, this is one of the few studies that has characterized epigenetic variation in an endangered species, which can improve our understanding of genomic and physiological changes to species being conserved through ex situ management.

As predicted, DNA methylation showed tissue‐type specificity, in congruence with other studies that analyzed whole blood samples alongside various other tissues (Derks et al., 2016; Husby, 2022; Schilling & Rehli, 2007; Zhou et al., 2017). While whole blood is routinely collected for health screenings of ex situ managed BFFs and is the most readily available biobanked tissue, our findings suggest that DNA methylation in blood may not show a strong association with variation in reproductive pathways. Gene networks built from DMclusters for blood and average litter size were not enriched for GO terms that annotated genes with known functionality in reproduction or development, in contrast to both our testes and sperm differential methylation analyses. While this is not entirely unsurprising due to the diverse functions of DNA methylation in mammalian white blood cells (Zhang et al., 2013), other studies have found blood‐based sampling sufficient to predict DNA methylation variation in connection to several phenotypes of interest in numerous taxa (e.g., *Papio cynocephalus*, Lea et al., 2016; *Tachycineta bicolor*, Taff et al., 2019), including breeding success (*Hirundo rustica*; Saino et al., 2017) and the onset of reproductive timing (*Parus major*, Viitaniemi et al., 2019; Lindner et al., 2021). Recent studies in humans have also found an increasingly promising relationship between DNA methylation and reproductive phenotypes, suggesting the possibility of future blood‐based assays to predict male infertility (Balasubramanian et al., 2019; Sarkar et al., 2019). Ultimately, genome‐wide correlation in methylation levels across tissue types may thus provide valuable information on individual‐level fitness variation, even if the tissues examined are not those most directly related to a particular phenotype. However, insights gained by examination of whole blood in the context of BFF reproductive success were limited in the present study.

Given these results (namely, differences observed in preliminary analyses conducted using all three tissue types and the known relevance of sperm morphology to male fecundity in BFFs), we focused downstream analyses on DNA methylation in part in testes and predominantly in sperm. As predicted, both tissues showed associations between differential DNA methylation and male reproductive phenotypes in gene networks enriched for reproduction and development. In testes, we found evidence via PCA clustering that DNA methylation variation may vary in binary, seasonal phenotypic changes (i.e., males increasing testes firmness during the breeding season) as they undergo rapid spermatogenesis to prepare for copulation and insemination of females (Hillman & Carpenter, 1984; Williams et al., 1991). Most seasonal breeders, including BFFs, shut down spermatogenesis in the testes outside the breeding season (Goeritz et al., 2003; Klonisch et al., 2006), although the epigenetic mechanisms behind many of these phenotypically plastic changes are poorly understood (Husby, 2022). At least one study has shown that epigenetic mechanisms do play a fundamental role in triggering molecular pathways in a seasonally breeding mammal by controlling photoperiod time measurement, which stimulates gonadal growth or regression (Stevenson & Prendergast, 2013). Furthermore, clinical studies and research in model systems continue to discover key links between epigenetic modifications in the testes and male infertility (Bunkar et al., 2016; Sadler‐Riggleman et al., 2019). We conclude that further exploration of testes samples is warranted in BFFs and could benefit from the use of single‐cell sequencing techniques (Guo et al., 2018). Such nuanced approaches might inform our understanding of spermatogenesis in ex situ BFFs beyond what we were able to discover from our analyses of bulk testes samples, which contain a mixture of cell types and can thus be implicated in a wide variety of gene functions.

Sperm was the tissue with the strongest observed associations between reproductive gene pathways and DNA methylation variation. Sperm had the highest per‐cytosine differential methylation and the highest number of significant cytosines associated with developmental and reproductive biological processes of the three tissues investigated. Note that the numerical disparity in the sheer number of DMS seen between sperm in comparison with other tissue types may be due in part to substantially higher rates of DNA methylation observed in sperm relative to other somatic tissues in human and non‐human mammals (Perrier et al., 2018; Zhou et al., 2018). Sperm samples specifically present logistical benefits to population managers, as semen collection is far less invasive than testes sampling, which relies on dissection during castration. The two gene networks constructed from sperm DMclusters associated with average litter size and sperm count were significantly enriched for biological functions directly related to reproduction and development and annotated numerous candidate genes with known relevance to reproductive regulation.

For example, *JARID2* is a transcriptional repressor known to induce epigenetic changes via its activity within the methyltransferase Polycomb repressive complex 2 (PRC2; Pasini et al., 2010). *JARID2* plays a key role in embryonic stem cell differentiation and normal development as a DNA‐binding protein that mediates the recruitment of the PRC2 complex to target genes in embryonic stem cells (Fischer et al., 2022; Margueron & Reinberg, 2011; Pasini et al., 2010). In addition, the gene encoding androgen receptor (*AR*) has been linked to androgen insensitivity syndrome, also known as testicular feminization syndrome (Shang et al., 2002); mutations in the *AR* gene complex are linked to increased risk of male infertility, oligospermia, hypospadias, and other transcription‐dependent reproductive abnormalities in males (Hiort et al., 2000; Keil et al., 2014). This gene (among other outliers we identified from gene network analyses, i.e., *ADAM12* and *EZH2*) is associated with transcriptional repression via epigenetic silencing, possibly suggesting that epigenetic changes which target these genes could affect regulatory activity by silencing gene expression in essential developmental pathways (Cao et al., 2002; Ray et al., 2011).

These findings are in alignment with similar studies in humans and domesticated animals (Costes et al., 2022; Rotondo et al., 2021; Štiavnická et al., 2022; Sujit et al., 2020; Takeda et al., 2021), which have all highlighted the critical role of DNA methylation in the proper development and functioning of sperm cells. Candidate genes that we identified from gene networks built from DMclusters in sperm are well‐studied and known to be involved in the regulation of developmental onset via epigenetic transcriptional repression in humans and mice, specifically in the multi‐protein complexes formed by *JARID2* and *EZH2* in PRC2 (Fischer et al., 2022; Margueron & Reinberg, 2011; Pasini et al., 2010). While genes within these complexes show a high degree of relevance to male fertility and have functionality that is potentially conserved, other genes identified in this study may play a lesser‐known role in transcriptional regulation that is yet to be understood. One caveat of DNA methylation studies in non‐model organisms is that expressional or functional validation data are often unavailable (Husby, 2020), so genes discovered in wildlife studies may not share any previously known biological functions, even when using the reference genome of a closely related species (Lindner et al., 2021).

As this study is association‐based, we recognize that changes to gene expression and the establishment of a causal link between DNA methylation and fertility declines require much further functional validation. However, the binomial modeling approach used here provides preliminary correlative information on associations between male reproductive phenotypes and potential molecular mechanisms for gene regulatory change in this species. The tools needed for experimental and functional validation of outliers identified from DNA methylation studies to specific phenotypes are not yet readily available for use in non‐model organisms such as BFFs (Romero & Lea, 2022). However, rapid technological advancements and an increase in publicly available genomic resources provide avenues for continued growth in our understanding of epigenetic variation in ex situ BFFs and other rare and threatened species. A scaffold‐level reference assembly of the BFF genome has recently become available (NCBI BioProject PRJNA634921), which, when further annotated and used in future studies, could increase our detection of genic, intergenic, or non‐promoter regions that may be methylation‐dependent. This is particularly relevant to our study, as, in contrast to our hypothesis, many DMS used in gene network analyses were widespread throughout the genome and not located solely within promoters, indicating that a diversity of genomic sites and contexts may be relevant to understanding the epigenetic basis of reproductive decline in ex situ BFFs.

Despite these limitations, results from this study demonstrate links between DNA methylation variation in sperm and two important male reproductive phenotypes, creating a tractable opportunity for future exploration of epigenetic variation in BFFs. For example, sperm obtained from electroejaculation presents an intermediate option between the ease of blood collection and the constraints of testicular tissue harvesting for BFF managers. Sperm can be collected annually from ex situ and wild (reintroduced) individuals during approved biomedical surveys. More frequent sperm sample collections could also be dually used to aid artificial insemination attempts and build up valuable inventory of cryopreserved samples (Santymire et al., 2014; USGS, 2005; Wisely et al., 2015), both of which directly support conservation managers in breeding and reintroduction goals. Future studies comparing reproductive phenotypes using DNA methylation in sperm collected from wild (reintroduced) and ex situ males would greatly improve our understanding of epigenetic pathway changes unique to the ex situ environment that may be driving the rise of maladaptive reproductive phenotypes, indicative of EDID.

In managing current and future BFF populations, the collection and cryopreservation of sperm from both ex situ and reintroduced BFFs will likely be paramount to understanding the epigenetic basis of male reproductive abnormalities, particularly as very low genetic diversity in the extant population continues to present unique challenges to ex situ breeding. Identifying differentially methylated CpGs associated with male fertility could support the downstream development of a targeted methylation sequencing panel that may predict the likelihood of greater reproductive success from methylation patterns (see Morselli et al., 2021) at select DMS for an individual breeder. Though requiring further research, the development of such an approach could more effectively guide management decisions regarding natural pairings and costly artificial insemination attempts, concentrating these resources on males with the highest observed sperm quality and anticipated fecundity. While we have yet to understand the direct role and relationship of DNA methylation with male fertility and full potential for EDID in ex situ managed BFFs, our results provide valuable baseline and logistical data (i.e., target tissue type) for BFF managers and conservation geneticists alike. We present evidence supporting the role of DNA methylation—and the need for its continued investigation—in the regulation of mammalian reproductive processes. These results provide new considerations to researchers and managers facing challenges in the conservation of the many endangered species reliant on human care for survival.

## 5 CONCLUSION

By integrating an evolutionary epigenetics framework into the conservation challenges confronting BFFs, we aimed to shed light on the epigenetic basis of the poor and declining reproductive health of ex situ animals. Within conservation genomics more broadly, this study increases our understanding of how epigenetic variation may contribute to environmentally driven phenotypic changes that have a direct impact on organismal fitness. Specific to BFFs, we identified the key sample type and associated male reproductive phenotypes most relevant to addressing the breeding challenges hampering the restoration of this endangered species to the landscape. BFFs represent an iconic example of large‐scale ex situ management; by improving our understanding of male fertility changes within human‐managed populations, we can improve the efficacy of national breeding programs and contribute to the continued success of wildlife conservation efforts for this and other species.

## FUNDING INFORMATION

This work was supported by the Smithsonian Institution Fellowship Program fellowship to ALD to support research conducted at the Center for Conservation Genomics at the Smithsonian's National Zoo and Conservation Biology Institute. RS was supported by the Association of Zoos and Aquariums Saving Animals From Extinction grant. Sample use was approved by the USFWS Black‐footed Ferret Recovery Program.

## CONFLICT OF INTEREST STATEMENT

The authors have no known conflicts of interest.

## DISCLAIMER

The findings and conclusions in this article are those of the author(s) and do not necessarily represent the views of the U.S. Fish & Wildlife Service.

## Supporting information


Appendix S1.

## Data Availability

Code used in analyses and figures can be found archived on GitHub (https://github.com/stavi‐t/black‐footed‐ferrets). Demultiplexed sequencing reads (BioSamples SAMN33193715 to SAMN33193738) have been deposited on NCBI SRA under BioProject PRJNA932458.
