# Supplementary material for: Epigenetic changes to gene pathways linked to male fertility in ex situ black‐footed ferrets
Source: Evol Appl. 2024 Jan 26;17(1):e13634. doi: 10.1111/eva.13634 (PMC10818088; doi:10.1111/eva.13634)
Supplement: Supplementary file 1 — Appendix S1. [file EVA-17-e13634-s001.docx]

**Table S1.** Sample name, studbook number, tissue type, sequencing library, sample ID, index primer number, index primer sequence, and number of demultiplexed reads for the 24 samples sequenced in this study.

| **Name** | **SB#** | **Tissue Type** | **Library** | **Sample ID** | **Index #** | **Index Sequence** | **Demultiplexed Reads** |
| --- | --- | --- | --- | --- | --- | --- | --- |
| Gale | 7611 | Testes | BFF1 | T2_1* | 1 | ATCACG | 36207454 |
| Jenson | 8519 | Sperm | BFF1 | S4_1* | 2 | CGATGT | 38170458 |
| Jenson | 8519 | Whole Blood | BFF1 | B4_1 | 3 | TTAGGC | 31157393 |
| Jenson | 8519 | Testes | BFF1 | T4_1 | 4 | TGACCA | 40066710 |
| Capone | 6536 | Sperm | BFF1 | S1_1 | 5 | ACAGTG | 34860482 |
| Padalecki | 8520 | Sperm | BFF1 | S5_1 | 6 | GCCAAT | 38435674 |
| Cane | 7685 | Testes | BFF1 | T6_1 | 7 | CAGATC | 37281795 |
| Flagstaff | 8161 | Whole Blood | BFF1 | B8_1 | 8 | ACTTGA | 38669442 |
| Pancake | 8393 | Whole Blood | BFF1 | B9_1 | 9 | GATCAG | 32550165 |
| Eamon | 7916 | Testes | BFF1 | T7_1 | 10 | TAGCTT | 43026127 |
| Padalecki | 8520 | Testes | BFF1 | T5_1 | 11 | GGCTAC | 34443078 |
| Tanis | 7419 | Whole Blood | BFF1 | B3_1 | 12 | CTTGTA | 32588215 |
| Tanis | 7419 | Sperm | BFF2 | S3_2 | 1 | ATCACG | 28510542 |
| Tanis | 7419 | Testes | BFF2 | T3_2 | 2 | CGATGT | 45664513 |
| Gale | 7611 | Testes | BFF2 | T2_2* | 3 | TTAGGC | 36461382 |
| Padalecki | 8520 | Whole Blood | BFF2 | B5_2 | 4 | TGACCA | 34356518 |
| Cane | 7685 | Whole Blood | BFF2 | B6_2 | 5 | ACAGTG | 34038839 |
| Flagstaff | 8161 | Testes | BFF2 | T8_2 | 6 | GCCAAT | 41524684 |
| Eamon | 7916 | Whole Blood | BFF2 | B7_2 | 7 | CAGATC | 30310711 |
| Pancake | 8393 | Testes | BFF2 | T9_2 | 8 | ACTTGA | 31684116 |
| Capone | 6536 | Testes | BFF2 | T1_2 | 9 | GATCAG | 34839543 |
| Jenson | 8519 | Sperm | BFF2 | S4_2* | 10 | TAGCTT | 40812128 |
| Gale | 7611 | Whole Blood | BFF2 | B2_2 | 11 | GGCTAC | 34896961 |
| Gale | 7611 | Sperm | BFF2 | S2_2 | 12 | CTTGTA | 31260558 |
| ** replicate sample* | |  |  |  |  |  |  |

**Table S2.** Sample identifier, number of cytosines (No. Sites), mean coverage, bisulfite conversion rate, and average ± standard deviation of methylation frequency across all CpG sites (Total) and each genomic motif (CG, CHG, and CHH) after mapping to the domestic ferret reference genome and filtering for ≥10X coverage.

|  |  |  |  | ***MC Average ± Standard Deviation*** | | | |
| --- | --- | --- | --- | --- | --- | --- | --- |
| **Sample ID** | **No. Sites** | **Coverage** | **Conv. Rate** | **Total** | **CG** | **CHG** | **CHH** |
| B2_2 | 18084334 | 27.251 | 99.414% | 0.100 ± 0.274 | 0.538 ± 0.414 | 0.003 ± 0.041 | 0.003 ± 0.039 |
| B3_1 | 16859239 | 23.992 | 99.374% | 0.097 ± 0.270 | 0.532 ± 0.414 | 0.003 ± 0.040 | 0.003 ± 0.038 |
| B4_1 | 15907341 | 26.021 | 99.482% | 0.098 ± 0.271 | 0.528 ± 0.414 | 0.003 ± 0.041 | 0.003 ± 0.038 |
| B5_2 | 17561369 | 26.203 | 99.425% | 0.094 ± 0.265 | 0.505 ± 0.416 | 0.003 ± 0.040 | 0.003 ± 0.037 |
| B6_2 | 17193824 | 27.106 | 99.449% | 0.093 ± 0.260 | 0.485 ± 0.407 | 0.003 ± 0.039 | 0.003 ± 0.037 |
| B7_2 | 15729758 | 25.272 | 99.376% | 0.103 ± 0.278 | 0.547 ± 0.414 | 0.003 ± 0.041 | 0.003 ± 0.039 |
| B8_1 | 19188807 | 28.544 | 99.439% | 0.097 ± 0.270 | 0.524 ± 0.414 | 0.003 ± 0.040 | 0.003 ± 0.038 |
| B9_1 | 16867999 | 26.167 | 99.478% | 0.092 ± 0.262 | 0.492 ± 0.416 | 0.003 ± 0.039 | 0.003 ± 0.037 |
| S1_1 | 17993096 | 23.886 | 99.402% | 0.099 ± 0.283 | 0.527 ± 0.453 | 0.003 ± 0.040 | 0.003 ± 0.040 |
| S2_2 | 14944112 | 23.646 | 99.413% | 0.102 ± 0.290 | 0.530 ± 0.459 | 0.003 ± 0.041 | 0.003 ± 0.041 |
| S3_2 | 13581877 | 21.643 | 99.372% | 0.093 ± 0.275 | 0.462 ± 0.459 | 0.003 ± 0.039 | 0.002 ± 0.039 |
| S4_1** | 19137267 | 25.323 | 99.470% | 0.100 ± 0.286 | 0.536 ± 0.456 | 0.003 ± 0.041 | 0.003 ± 0.040 |
| S4_2* | 19723685 | 26.453 | 99.400% | 0.101 ± 0.287 | 0.529 ± 0.457 | 0.003 ± 0.041 | 0.003 ± 0.041 |
| S5_1 | 19068100 | 26.323 | 99.436% | 0.101 ± 0.289 | 0.559 ± 0.455 | 0.003 ± 0.041 | 0.003 ± 0.040 |
| T1_2 | 18223430 | 20.041 | 99.343% | 0.088 ± 0.251 | 0.460 ± 0.402 | 0.003 ± 0.036 | 0.003 ± 0.036 |
| T2_1** | 21557074 | 22.433 | 99.406% | 0.092 ± 0.257 | 0.483 ± 0.403 | 0.003 ± 0.037 | 0.003 ± 0.037 |
| T2_2* | 20720072 | 21.322 | 99.235% | 0.092 ± 0.257 | 0.468 ± 0.404 | 0.003 ± 0.037 | 0.003 ± 0.038 |
| T3_2 | 23991210 | 22.485 | 99.375% | 0.096 ± 0.266 | 0.495 ± 0.415 | 0.003 ± 0.038 | 0.003 ± 0.039 |
| T4_1 | 21043527 | 22.999 | 99.408% | 0.077 ± 0.230 | 0.404 ± 0.386 | 0.003 ± 0.035 | 0.003 ± 0.034 |
| T5_1 | 19534683 | 22.675 | 99.362% | 0.089 ± 0.250 | 0.456 ± 0.398 | 0.003 ± 0.037 | 0.003 ± 0.037 |
| T6_1 | 21272596 | 22.346 | 99.331% | 0.085 ± 0.243 | 0.461 ± 0.390 | 0.003 ± 0.035 | 0.003 ± 0.036 |
| T7_1 | 24299800 | 25.249 | 99.371% | 0.097 ± 0.268 | 0.519 ± 0.413 | 0.003 ± 0.038 | 0.003 ± 0.039 |
| T8_2 | 22817312 | 24.872 | 99.359% | 0.097 ± 0.268 | 0.511 ± 0.413 | 0.003 ± 0.038 | 0.003 ± 0.039 |
| T9_2 | 16953150 | 22.353 | 99.340% | 0.098 ± 0.271 | 0.503 ± 0.418 | 0.003 ± 0.038 | 0.003 ± 0.039 |

** replicate sample ** replicate retained for downstream analysis*

**Figure S1.** Average methylation frequency (MF) for all cytosines (Total) and each genomic motif (CG, CHG, and CHH) for blood, sperm, and testes samples included in this study.

**­**

**Figure S2.** Principal component analysis (PCA) of all 24 samples showed strong overlap between positive control samples sequenced across libraries. Downstream analyses therefore excluded replicates and only included 22 unique samples from nine individuals.

**Figure S3.** PCA plots showing a) PC2 and PC3 and b) PC3 and PC4 exhibit clustering by sample type. Axes indicate percent of variation explained by the principal components.

**Table S3.** Associations between metadata categories and the first five principal components (PC) identified using reads mapped to the domestic ferret genome. We used Kruskal-Wallis (KW) tests for the categorical metadata variables: sample type, ferret identifier, and library batch identifier, and provide the chi-squared test statistic (*var*) and significance value (*p*). We used Pearson’s product-moment correlation for the continuous metadata variable bisulfite conversion rate and similarly provide the *t* test statistic (*var*) and significance value (*p*). Degrees of freedom (*df*) for each test are provided, and *p-*values passing the significance threshold of 0.05 are indicated with an asterisk.

|  | **Sample Type (df=2)** | | **Ferret ID (df=8)** | | **Batch ID (df=1)** | | **BS Conv. (df=20)** | |
| --- | --- | --- | --- | --- | --- | --- | --- | --- |
|  | *var* | *p* | *var* | *p* | *var* | *p* | *var* | *p* |
| *PC1* | 16.821 | <0.001* | 3.755 | 0.879 | 0.109 | 0.742 | 2.088 | 0.050* |
| *PC2* | 18.344 | <0.001* | 3.146 | 0.925 | 0.352 | 0.553 | 1.193 | 0.247 |
| *PC3* | 18.344 | <0.001* | 2.123 | 0.977 | 0.352 | 0.553 | -2.994 | 0.007* |
| *PC4* | 6.351 | 0.042* | 7.285 | 0.506 | 3.170 | 0.075 | -1.480 | 0.155 |
| *PC5* | 4.085 | 0.130 | 5.929 | 0.655 | 0.039 | 0.843 | 0.640 | 0.530 |

**Table S4.** Significant differentially methylated sites (DMS) and constructed DMclusters associated with the reproductive phenotypes average litter size (blood, testes, sperm), sperm count (sperm), and testes firmness (testes) from the five binomial mixed models.

| **Tissue** | **Phenotype tested** | **No. DMS** | **No. Sig. DMS** | **No. Sig. DMclusters** |
| --- | --- | --- | --- | --- |
| Blood | Average litter size | 9427357 | 56487 | 30373 |
| Testes | Average litter size | 9118485 | 52510 | 28727 |
| Sperm | Average litter size | 8936345 | 83024 | 44199 |
| Sperm | Sperm count | 9494233 | 84581 | 43261 |
| Testes | Testes firmness | 7616790 | 36151 | 12140 |

**Figure S4.** Raw site annotation counts for differentially methylated sites (DMS) and their categorical genomic locations identified in results from binomial mixed models for blood, testes and sperm in the five separate analyses: **A**. Average litter size in blood, **B**. Average litter size in sperm, **C**. Average litter size in testes, **D**. Sperm count in sperm and **E**. Testes firmness in testes.

**Figure S5.** Gene networks constructed using the *STRING* plugin v1.4.2 in *Cytoscape* v3.9.1 for the five separate gene network analyses of: **A**. Average litter size in blood, **B**. Average litter size in testes, **C**. Average litter size in sperm, **D**. Sperm count in sperm and **E**. Testes firmness in testes. These gene networks were built from analysis of differential DNA methylation at clusters of three or more DMS. Colors indicate associated gene IDs.

**Figure S6.** Quantile-quantile (QQ) plots of -log10(p-values) from a theoretically expected uniform distribution compared to p-values generated from binomial mixed models of DNA methylation count data. Observed p-values from the five separate analyses are shown: **A**. Average litter size in blood, **B**. Average litter size in sperm, **C**. Average litter size in testes, **D**. Sperm count in sperm and **E**. Testes firmness in testes. Samples sizes vary between analyses (see main text). All models include age at sampling and pedigree-based relatedness estimates as covariates.
